# Effect of Nano Spinel Ferrites Co_0.9_Cu_0.1_Fe_2_O_4_ on Non-Isothermal Cold Crystallization Behaviours and Kinetics of Its Composites with Polylactic Acid

**DOI:** 10.3390/polym16091190

**Published:** 2024-04-24

**Authors:** Wael H. Alsaedi, Khulood A. Abu Al-Ola, Omaima Alhaddad, Zyzafon Albelwe, Renad Alawaji, Ahmed M. Abu-Dief

**Affiliations:** 1 Department of Chemistry, College of Science, Taibah University, Al-Madinah Al-Munawarah P.O. Box 30002, Saudi Arabia; 2 Department of Chemistry, Faculty of Science, Sohag University, Sohag 82524, Egypt

**Keywords:** PLA, Co_0.9_Cu_0.1_Fe_2_O_4_ magnetic nanoparticles, hydrothermal synthesis, crystallization, POM, DSC, TGA

## Abstract

Nanoparticles of spinel ferrites with a composition of Co_0.9_Cu_0.1_Fe_2_O_4_ (AM NPs) were effectively synthesized via a hydrothermal route. The structure of ferrite nanoparticles was characterized with X-ray diffraction, which showed a single cubic spinel phase. Energy-dispersive X-ray (EDX) spectroscopy and field emission-scanning electron microscopy (FE-SEM) were employed to analyse elemental composition and surface morphology, respectively. Moreover, the effects of the Co_0.9_Cu_0.1_Fe_2_O_4_ on the morphology of [PLA = polylactic acid] nanocomposites were examined through polarized light optical microscopy (POM) and X-ray diffraction (XRD). The thermal behaviours for tested samples were studied through [DSC = differential scanning calorimetry] and [TGA = thermal gravimetric analysis]. A great number of minor PLA spherulites were detected using POM in the presence of the Co_0.9_Cu_0.1_Fe_2_O_4_ ceramic magnetic nanoparticles (AM), increasing with AM nanoparticle contents. X-ray diffraction (XRD) analysis showed that the presence of nanoparticles led to an increase in the intensity of diffraction peaks. The DSC findings implied that the crystallization behaviours for the efficient PLA as well as its nanocomposites were affected by the addition of AM nanoparticles. They act as efficient nucleating agents because they shift the temperature of crystallization to a lower value. The Avrami models were used to analyse kinetics data. The experimental data were well described using the Avrami method for all samples tested. The addition of AM to the PLA matrix resulted in a decrease in the crystallization half-time t_1/2_ values, indicating a faster crystallization rate. TGA data showed that the occurrence of AM nanoparticles decreased the thermal stability of PLA.

## 1. Introduction

Special physicochemical characteristics of spinel ferrite nanoparticles include substantial magnetic crystalline anisotropy, a high Curie temperature, good thermal stability, and an acceptable chemical composition. Furthermore, they possess outstanding magnetic properties, may be easily manipulated in terms of size and form, have a high specific surface area, surface active sites, good chemical stability, and are easily functionalized or changed [1,2].

In recent years, biopolymers have drawn significant interest worldwide due to their promising future in packaging for food, therapeutic uses, and environmentally friendly polymer substances [3,4,5,6]. Polylactic acid (PLA) is a significant polyester that degrades naturally and is made of renewable resources. It is a promising green material because it comprises raw material, lactic acid, and can be efficiently produced by fermentation from renewable resources. It has mechanical qualities that are similar to those of conventional thermoplastic materials [3]. But, because of its essential slow crystallizing rate, influenced PLA objects are typically amorphous according to fast cooling operations, like [IM = injection moulding] [3,4,5,6,7]. This limits the use for PLA to be a material for engineering since the modulus as well as thermal resistance for the amorphous items drop considerably above its temperature at which glass transitions at 60 °C [6,7,8]. Crystallization plays a crucial role in determining the properties and performance of polylactic acid. By controlling the crystallization process, it is possible to enhance the mechanical strength and thermal stability of polylactic acid. This can be achieved through the addition of a nucleating agent, which promotes the formation of crystalline structures in the polymer. The use of a nucleating agent can maximize the crystallization of polylactic acid, leading to improved material properties and performance [9]. Additionally, slow crystallizing typically leads to longer moulding cycles and raises part ejection. Several possible nucleating means were investigated, involving glass fibres, talc, a halloysite nanotube, and nano (TiO_2_, Ag, ZnO) and phenyl phosphonic acid zinc [10,11,12,13,14,15,16,17]. The most famous nucleating agents that have been found to increase the degree of crystallinity of polylactic acid are talc, montmorillonite, and carbon nanotubes. These nucleating agents act as heterogeneous nucleating sites, promoting the formation of ordered crystal structures in PLA and enhancing its overall crystallinity. Additionally, studies have also shown that boron nitride, nano fillers, ethylene bis-stearamide, and LAK are also effective nucleating agents for polylactic acid [18]. For instance, the maximum percentage of crystallization of polylactic acid in the presence of talc is 42% [19]. There are several techniques for developing ceramic nanoparticles with magnetic properties in a spinel form, involving hydrothermal, sol-gel, microwave-assisted, thermal degradation, solvothermal, and co-precipitation [20,21,22,23,24,25]. The selection of the approach of preparation is based on various parameters, the maximum essential characteristics of substance components, the wanted particle size, and the treatment in which the compound is applied. Similarly, an improvement in preparation factors is one of the actual valuable factors in achieving the required compound.

Nanocomposites of ferrite with PLA can improve the mechanical properties of PLA, including tensile strength, modulus, and toughness. Ferrite nanoparticles act as reinforcing fillers, enhancing the structural integrity and performance of PLA-based materials for applications in automotive, aerospace, packaging, and construction industries. Here, we utilized the hydrothermal approach to produce Co_0.9_Cu_0.1_Fe_2_O_4_ (AM NPs) due to the advantages for this technique, like, particle size control, colloidal stability, stoichiometric composition, morphology, and uniformity/crystallinity quality for AM NPs [26].

This study expected to synthesise and investigate composites of PLA with Co_0.9_Cu_0.1_Fe_2_O_4_ (AM NPs) as a novel nucleating agent on the non-isothermal cold crystallization behaviour of PLA, which is expected to improve the crystallization of PLA more than any reported material in the literature [10,11,12,13,14,15,16,17,18,19].

The crystallization performance of PLA was examined through polarized optical microscopy (POM), TGA, and DSC.

## 2. Experimental Methods

### 2.1. Materials

All chemicals like Et-OH and Acetone employed in this study were all of high analytical quality, not including any extra purification. (Copper, cobalt, and iron) nitrates (Sigma-Aldrich, Darmstadt, Germany) were precursors for [Co (II), Cu(II), and Fe(III)] metal ions. NaOH pellets acted as the precipitating agent (Sigma-Aldrich), and surfactant was polyethylene glycol 400 (Sigma-Aldrich). Each solution, including the stoichiometric amounts for the metal salts under study, was made with deionized water.

The PLA consisted of [98% of L-lactic acid with 2]% of D-lactic acid and was provided from (Biomer Company; Krailling, Germany) through a profitable mark well known as (Biomer L9000), an average M.wt of 200 kDa, and an index of polydispersity (1.98). After being delivered as white pellets, the PLA was used right away without needing to be further dried. The 99.9% pure chloroform was obtained from Sigma-Aldrich in Germany.

### 2.2. Preparation of the Investigated Nano Oxides

**Figure 1:**
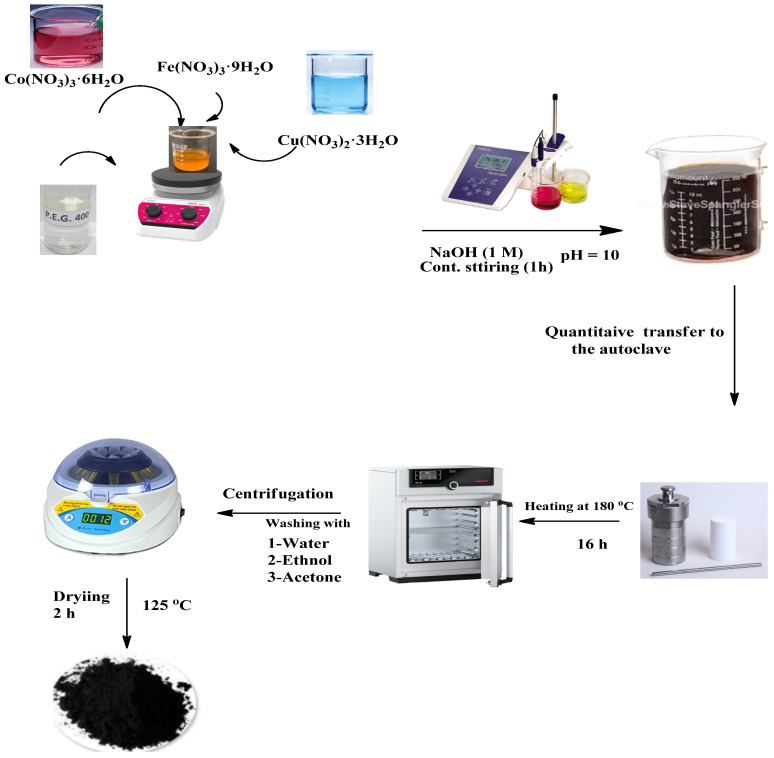
Pathway diagram of hydrothermal synthesis process of Co_0.9_Cu_0.1_Fe_2_O_4_ nanoparticles.

Copper-doped cobalt ferrite nanoparticles were prepared using an equal stoichiometric quantity of [Cu(II), Co(II), and Fe(III)] nitrate salts that were dissolved in [30 mL] of H_2_O. The final mixture was stirred with a magnetic agitator until all the metal salts from the precursor material were dissolved in deionized H_2_O. Then, 5 mL, PEG-400 was enhanced drop by drop to the resulting mixture, which was supposed to act as a surfactant to cover the prepared metal oxide nanoparticles and prevent them from accumulating together. For the next 30 min, the resulting solution was continuously stirred. Next, the pH level of the solution was modified to 10 by gradually adding a drop of NaOH (1 M) solution. After 1 h of constant stirring, a homogenous solution that included the metal salts’ hydroxide-form precipitates was generated. Finally, the resulting mixture (80 mL total volume) was sealed in stainless Teflon-lined autoclaves, heated to 180 °C for 16 h, and then slowly cooled gradually to room temperature. After centrifugation, the resultant products were repeatedly washed with (deionized H_2_O, EtOH, and acetone) before being dried for two hours at 125 °C. The steps required to synthesize the examined metal oxide nanoparticles are described in Figure 1.

### 2.3. Characterizations of the Investigated Nano Oxides

The prepared structural factors, phase verification, and chemical composition of the Co_0.9_Cu_0.1_Fe_2_O_4_ sample were inspected through [XRD = X-ray diffraction, Philips PW3040, Portland, ON, USA] and [EDXS = energy-dispersive X-ray spectroscopy, JEOL-6610VL, Tokyo, Japan]. Using a [TEM = transmission electron microscope, JEOL-JEM-2100, Tokyo, Japan] and [SEM= scanning electron microscope, JEOL-6610VL, Tokyo, Japan] analyser, the surface-form microscope slides and the distribution of particle sizes of the studied nano spinel ferrites were completely exposed. Using FT-IR (PerkinElmer, Waltham, MA, USA) the phase verification and chemical quality of the materials under study were confirmed.

Magnetic behaviour was inspected through a SQUID-magnetometry (Portland, ON, USA) technique around areas up to [±40 KOe].

### 2.4. Preparation of the PLA Nanocomposites

The casting film method was applied to synthesize combinations of PLA through various nano oxide contents. For all transparent reports, about 2.0 g of the total tested sample was merged and placed in 50 mL of chloroform and stirred around 40 °C for 45 min to assure full-section dissolution. The solution was transferred to a glass Petri dish and left there until the solvent gradually evaporated and uniform films were formed. The blends’ specifics are listed in Table 1.

### 2.5. Characterization of the PLA Nanocomposites

The spherulitic structures for the studied samples were investigated through an Olympus CX-31 (Tokyo, Japan) (POM) equipped with a numerical camera process (E330, Tokyo, Japan). Tested samples weighing [3:5 mg] were heated up on a glass slide via a cover slide and were melted onto a glass slip with a cover slip to form [20:50 μM] thick films. Every sample was heated around 200 °C for 180 s on a hot phase and cooled to the chosen crystallization rate of 110 °C. Polarized light photographic microscopy was performed following a 1 h annealing process at 110 °C crystallization temperature. The formation of crystals was studied through crossed polarizers. Multiple images were taken from a sample to avoid localization effects. The Shimadzu XRD-6100 (Portland, ON, USA) X-ray diffractometer was deployed to measure wide angle X-ray diffraction (WAXD) employing Cu-Kα radiation (λ = 0.154 nm, 40 kV, and 30 mA). The 2θ range of 10°–120° was applied to record the X-ray diffraction patterns at room temperature, with a scanning step and rate of 0.02° and 2° min^−1^, respectively. DSC-Q2000 (TA Instruments, New Castle, DE, USA) was used to determine thermal properties. Employing a Universal Analysis 2000 as well as TA Instruments Co.’s refrigerator cooling system RCS90 (Tokyo, Japan), the DSC-Q2000 was employed to evaluate the samples’ transformation temperatures. Indium was used for calibrating DSC with respect to heat flow according to the previously described methodology [27]. Each study was carried out in a N_2_ atmosphere [30 mL/min]. During the initial heating operate, the tested samples were firstly heated at [0 °C:200 °C] around for [10 °C/min] to dissipate their thermal properties and improve the contact between the surfaces; then, they were cooled to [0 °C] at [10 °C/min] and were reheated up to [200 °C] at a rate of [10 °C/min] in the second heating cycle. The results for the initial heating operation are not examined. The [*T_g_* = glass transition temperature] was evaluated as the intermediate of the thermal capacity stage during the second heating cycle. [(*T_cc_* and *T_m_*) = (cold crystallization and melting temperature], and their [Δ*H_cc_* and Δ*H_m_*] enthalpies were estimated through individual (exothermal and endothermal) processes via DSC during the second heating cycle. The [*X_c_* = overall crystallinity of PLA] was estimated with

(1) $$X_{c}=\frac{{∆H}_{cc}}{{∆H}_{m}^{{}^{\circ}}\cdot w}\times 100$$

[Δ*H_m_* and Δ*H_cc_*] represent the investigational [melting and cold] crystallization enthalpy, respectively. [*w*] represents the weight portion of PLA through its blend, and [${∆H}_{m}^{{}^{\circ}}$] represents the melting enthalpy for the crystalline structure of PLA (100%), which is accepted as 93.6 J/g [28]. TGA was carried out using TA machines [SDT-Q600, Tokyo, Japan]. The used sample weight was around [2:5 mg], sited in an alumina crucible, and was subjected to a heating ratio of [10 °C/min] from [25:700 °C]. The tests were performed in a N_2_ atmosphere via a removal rate of [20 mL/min] to eliminate thermal oxidative decomposition. The characteristics of initial thermal decomposition temperatures (*Ti*) after 10% weight loss and the complete decomposition temperature (*Tf*) of the samples were detected from the TGA curve. Both PLA and PLA/AM composites were heated twice for the kinetics investigation, with the first cycle taking place at a temperature ranging from 0 to 200 °C at a heating rate of 60 °C/min. The samples were maintained in isothermal conditions at 200 °C for 5 min. Thereafter, the samples were allowed to cool at a rate of 60 °C/min. The samples were maintained in an isothermal state at 0 °C for 5 min. DSC analysis for PLA and PLA/THB composites was also conducted at various heating rates (2.5, 5.0, 7.5 and 10 °C/min) to study non-isothermal cold crystallization kinetics.

## 3. Findings and Discussion

### 3.1. X-ray Diffraction (XRD) Analysis for the Prepared Co_0.9_Cu_0.1_Fe_2_O_4_ Nano Spinel Ferrite

An XRD evaluation was utilized to analyse and verify the cubic spinel stage structure and the micro-structural factors for the tested Co_0.9_Cu_0.1_Fe_2_O_4_ nano spinel ferrite. Figure S1 describes the graphic interpretation for XRD diffractograms for the prepared Co_0.9_Cu_0.1_Fe_2_O_4_-matched nano spinel ferrite. As explained in Figure S1, the examined sample has a poly-oriented state. Eight diffraction peaks were observed for the explored sample relating to the Miller indices [i.e., (hkl) planes as (111); (220); (311); (222); (400); (442); (551), and (440)]. The position of the line agrees through reference result JCPDS PDFcard No. 022-1086 to the [FCC = face-centred cubic] spinel structure of pure CoFe_2_O_4_ through the $Fd\bar{3}$m universe group [29]. There is no proof of a resulting impurity period for x ≤ 0.10. This confirms that the exchanged Cu^2+^ ions are absolutely dispersed in the cobalt ferrite spinel association structure. The studied crystallite sizes representing the tested Co_0.9_Cu_0.1_Fe_2_O_4_ from 11.3 nm were obtained in accordance with Sherrer’s relation [30,31,32,33].

### 3.2. Electron Microscopy Analyses Using EDXS, SEM, and TEM

EDXS is one of the greatest significant techniques that permitted the researchers of this paper to confirm the concentrations of the tested compounds. Furthermore, it signifies the most common method in verifying the exact doping proportions for the nano-spinel-ferrite-mixed molecule [34]. So, this method was applied to verify the high purity grade of our inspected samples and to verify the % of Cu^2+^ involved on the ferrite-mixed molecule. 

For the Cu_0.1_Co_0.9_Fe_2_O_4_ sample, just four spectral bands with a relationship to Co, Cu, Fe, and O_2_ elements denote the high grade of purity for the studied hydro-thermal compounds. To attain the accurate % of Cu content, we performed an analytical elemental study for Cu_0.1_Co_0.9_Fe_2_O_4_-mixed nano spinel ferrites, as shown in Table S1. The data proved that the [Cu/Co] relation for the tested Cu_0.1_Co_0.9_Fe_2_O_4_ sample is equivalent to its minimal stoichiometry.

**Figure 2:**
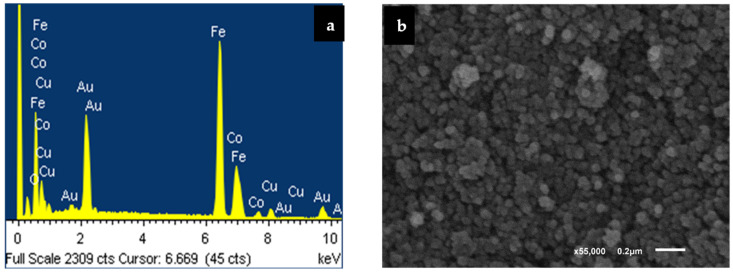
(**a**) Energy-dispersive X-ray spectroscopy (EDXS) spectra and (**b**) SEM micrograph of the investigated Cu_0.1_Co_0.9_Fe_2_O_4_ nanoparticles.

**Figure 3:**
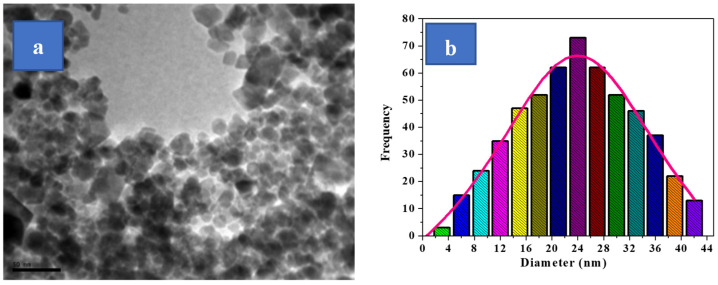
(**a**) TEM micrograph and (**b**) histogram of particle size distribution of the studied [Cu_0.1_Co_0.9_Fe_2_O_4_] nanoparticles.

Figure 2 exhibits an SEM graph for the sample tested for Cu_0.1_Co_0.9_Fe_2_O_4_ nano spinel ferrite. The SEM study verified the nano-sized circular modelled particle structure for the examined nanoparticles through a grain size that changed around 50 nm, which cannot be beneficial and depend on SEM analysis for evaluating the sizes of the tested particles that comprise the substance, due to the low level of the particle-form resolution calculated through this method. Here, a group of crystals can appear to be the same single crystal, creating an inaccurate impression about the material’s particle size. However, the magnetic interaction between the particles of magnetic substances, which leads to the fragments seeming to be like aggregates, had an important effect on this approach. This makes it impossible to determine particle size precisely. Regarding these explanations, we employed a TEM microscope, one of the most exact instruments within this field, to investigate the crystallite dimensions of the Cu_0.1_Co_0.9_Fe_2_O_4_-mixed nano spinel ferrite samples. TEM images of the Co_0.9_Cu_0.1_Fe_2_O_4_-mixed nano spinel ferrite sample are presented in Figure 3. The spherical forms across all the particles are uniformly sized and connect with the micrographs from SEM. We used the free program Image J for analysing more than 120 particles from the sample under study (Figure 3). Examine the variation in particle size and obtaining accurate results of the dimensions of particles is vital for understanding several physical characteristics of ferrites. The diameters of the particles estimated using XRD and TEM results are equivalent to about 24 nm, corresponding to the particle size distribution graph.

**Figure 4:**
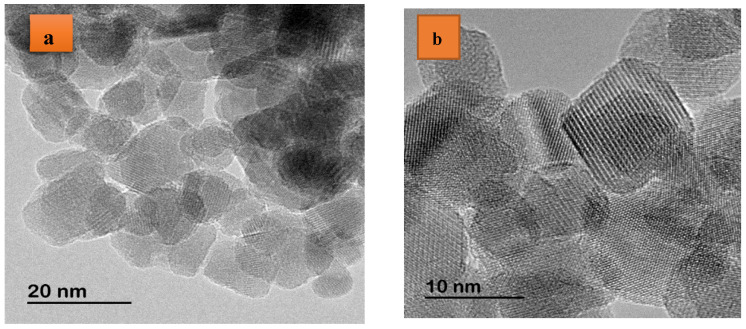

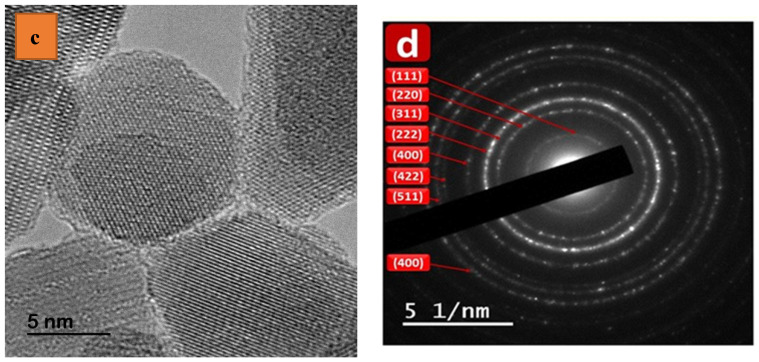
(**a**–**c**) HRTEM micrographs at several scales; (**d**) SAED models for Cu_0.1_Co_0.9_Fe_2_O_4_ nanoparticles.

HRTEM micrographs at different levels of pure Cu_0.1_Co_0.9_Fe_2_O_4_ nanoparticles, shown in Figure 4a–c, respectively, clearly exhibit the high crystalline nature of these nano systems. [SAED = selected area electron diffraction] patterns of pure Cu_0.1_Co_0.9_Fe_2_O_4_ nanoparticles shown in Figure 4d illustrate the polycrystalline nature of the sample. All diffraction rings were indexed to the [(111); (220); (222); (311); (400); (422); (511)] and (400) planes that fit into the space group *Fd-3m*. HRTEM and SAED studies are in good agreement with regard to the XRD data.

### 3.3. Analysis of Infrared Spectroscopy

Generally, in the far-infrared region, i.e., 350–600 cm^−1^, for all materials with a spinel ferrite structure, there are two or three main metal/oxygen absorption bands that are seen in the FT-IR spectrum. High-frequency peaks are detected at a wavenumber area of 500–600 cm^−1^, and low-frequency peaks are typically detected at a wavenumber area of 350–500 cm^−1^ [35,36]. In accordance with Waldron, these peaks agree with [A and B] sublattice site vibrations, separately [37]. The entrance of such interesting lines proves the configuration of the studied samples’ spinel ferrite framework.

Figure S2 displays the FT-IR transmission spectrum within a wavenumber scale of 400 and 4000 cm^−1^ for Cu_0.1_Co_0.9_Fe_2_O_4_ nano systems, which is in the FT-IR range of the spectra for all tested samples that has three specific metal/oxygen bond vibrations labelled υ_1_, υ_2_, and υ_3_. The lower frequency peaks (υ_1_ = 415 cm^−1^) and (υ_2_ = 480 cm^−1^) are recognized by bivalent tetrahedral A-site metal-O_2_ group chelates and extending vibrations for tri-valent metal/oxygen (Fe^3+^–O^2−^) bonds within an octahedral B-site, respectively [38]. The higher frequency peaks (υ_3_ = 587 cm^−1^) are associated with an extending tri-valent metal/oxygen (Fe^3+^–O^2−^) bond vibration in an octahedral B-site structure [39].

### 3.4. Magnetic Properties

**Figure 5:**
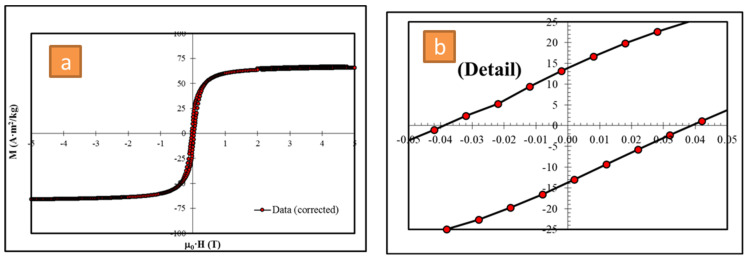
(**a**) Magnetization field (M-H) and (**b**) magnification of the centre part of hysteresis loops for the studied Cu_0.1_Co_0.9_Fe_2_O_4_ nanoparticles at 300 K.

The magnetization of the M–H loops to Cu_0.1_Co_0.9_Fe_2_O_4_ nano spinel ferrite tests at 300 K, and over a broad range of employed magnetic fields, is presented in Figure 5. The sample in this study illustrates a soft ferromagnetic nature, and the variety in oscillation forms indicates why the addition of the Cu^2+^ material influences the sample’s magnetic characteristics. From Figure 5, it can be seen that the magnetic settings were determined and associated with Cu^2+^-doping content being present. These variables involved the saturation remanence (Mr = 17 emu/g), (Ms = 66 emu/g) magnetic field, squareness (Mr/Ms = 0.26), coercivity (Hc; 475 Oe), magnetic moment (nB = 2.75 μB), and anisotropy parameter (k; 31.89 × 10^3^ erg. Oe/g). Several researchers have demonstrated that there is a significant association between the Ms values of spinel nanoparticles made of ferrite and the particle size of the prepared sample [40,41,42,43].

### 3.5. Polarized Light Optical Microscopy (POM)

**Figure 6:**
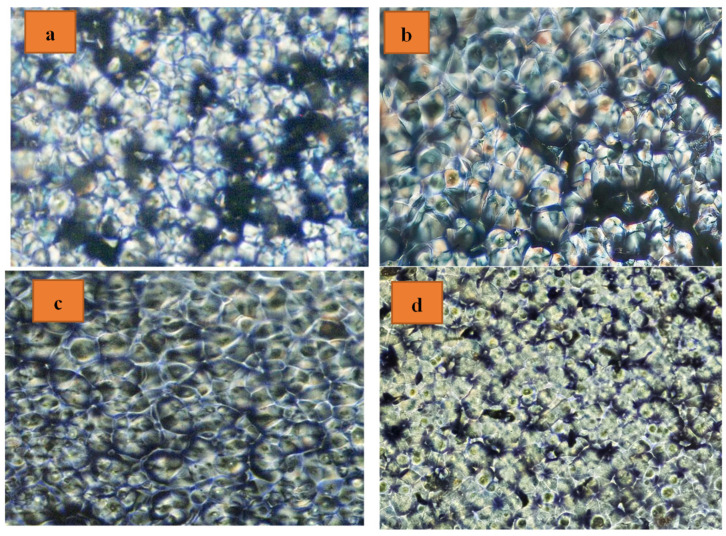
Polarized optical micrographs of (**a**) PLA spherulites, (**b**) PLA99.5/AM0.5, (**c**) PLA99/AM1, and (**d**) PLA98/AM2 nanocomposites with ratios of 100:0, 99.5:0.5, 99:1, and 98:2, after isothermal crystallization at 100 °C.

Figure 6 signifies the POM of the PLA and PLA/AM nanocomposites around the isothermal crystallization phase at 100 °C. The data exposed that the presence of Co_0.9_Cu_0.1_Fe_2_O_4_ ceramic magnetic nanoparticle fractions were attracted to the fabrication of little spherulites more than that occurring in the pure PLA. Also, growing the quantity of the AM similarly improved the quantity of small spherulites. According to these findings, AM is among the PLA’s most highly effective nucleating agents.

### 3.6. X-ray Diffraction (XRD) Analysis

**Figure 7:**
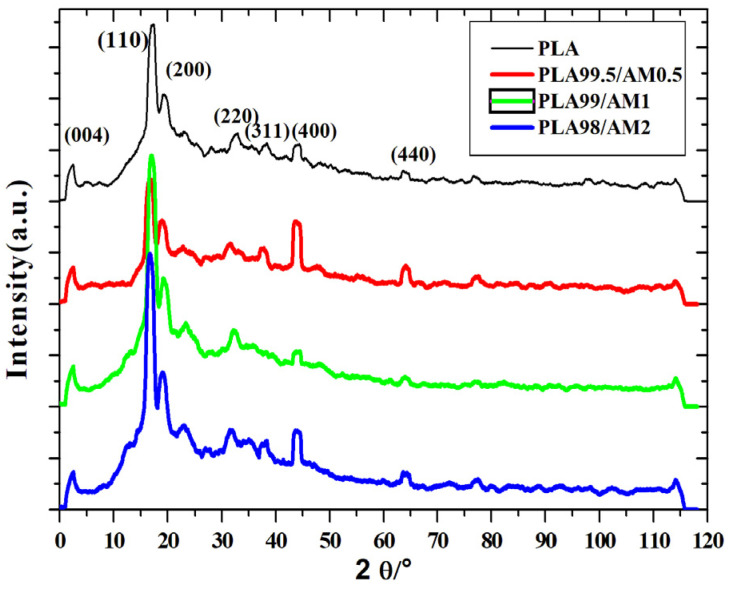
X-ray diffraction (XRD) lines of the PLA and its nanocomposites.

Figure 7 demonstrates the X-ray diffraction structures of pure PLA as well as its nanocomposites. The nanocomposites that were studied displayed identical diffraction peaks, suggesting that the PLA structure of the crystals is unmodified. To put it briefly, the PLA’s structure of crystals stayed unaffected due to Co_0.9_Cu_0.1_Fe_2_O_4_ ceramic magnetic nanoparticles. Furthermore, as Figure S1 illustrates, pure nanoparticles were extremely crystalline. However, the PLA nanocomposites did not display the usual peak patterns of nanoparticles; this is believed to have occurred since there are several locations for PLA to nucleate and because of the crystalline PLA particles that could have been formed during the cold crystallizing technique. Furthermore, the magnitude of the peaks in diffraction increased with the presence of nanoparticles. Consequently, nanoparticles indicated an efficient nucleating agent effect in the PLA matrix.

### 3.7. Thermal Gravimetric Analysis (TGA)

**Figure 8:**
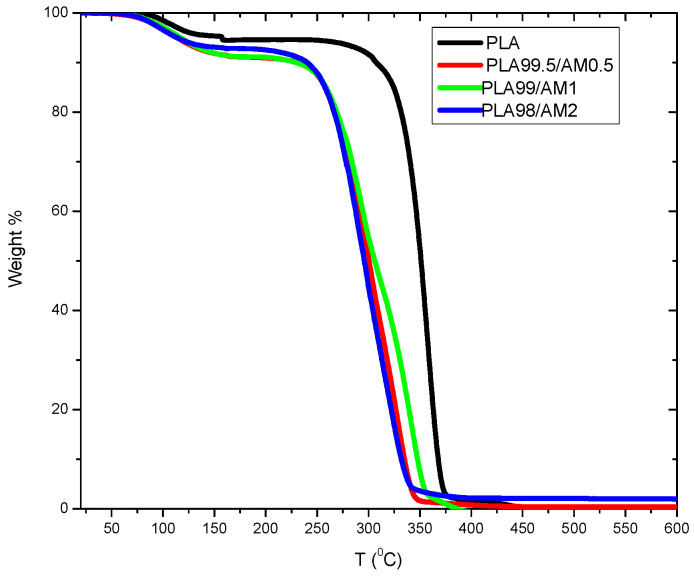
Thermogravimetry curves of PLA/AM composites.

The behaviour of thermal stability for PLA in the structure was evaluated through TGA, as represented in Figure 8 and as listed in Table 2. The highest decomposition temperature for the natural PLA was accomplished at 371 °C. Oppositely, the PLA/AM structures degraded with a one-stage decomposition pathway, which was established to be reduced more than that of the natural PLA through [356–335 °C]. Also, the thermal decomposition temperatures for PLA/AM composites were nearly constantly equal to 2% for AM, as outlined in Table 2. These obtained data indicate which = Co_0.9_Cu_0.1_Fe_2_O_4_ AM NPs caused the decomposition of the tested PLA. This may be attributed to the catalytic effect of the AM, which can reduce the thermal stability of PLA.

### 3.8. Differential Scanning Calorimetry (DSC)

#### 3.8.1. Non-Isothermal Cold Crystallization Behaviours of PLA/AM Composites

**Figure 9:**
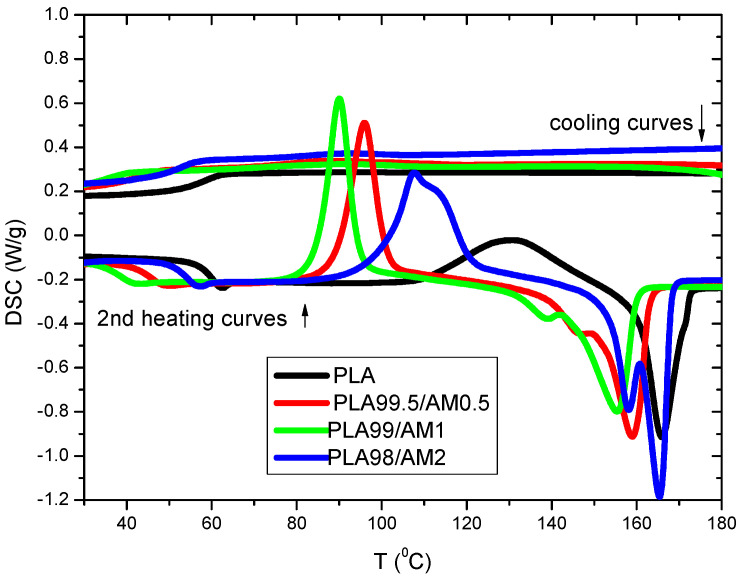
DSC curves of PLA/AM composites at a heating rate 10 °C/min.

It is recognized that thermal conversions for polymers have major influence on their uses as well as processing features [5,6,7,8,9]. So, it is important to examine the result of the Co_0.9_Cu_0.1_Fe_2_O_4_ AM NP composition on the crystallization rate, melting, and crystallinity of PLA. DSC cycle runs (heat–cool–heat) were performed for PLA composites. The first heating runs were conducted to improve the thermal contact between samples and DSC pans and to remove the thermal history of the sample; therefore, the results of the first DSC runs were not discussed. No exothermal crystallizing band was detected for the PLA/AM tested samples. The charted data (Figure 9) specified which studied PLA samples are amorphous through the rate of cooling per [10 °C/min]; moreover, the existence of AM might not suggestively modify the tested PLA crystallization rate when the studied PLA melt is cooled per [10 °C/min]. Figure 9 signifies DSC 2nd heating curves for PLA/AM composites. All parameters resulting from these curves are listed in Table 3. For PLA/AM composites, broad weak glass transition temperatures, with a slight change in heat capacities, were detected for all tested composites. This behaviour is in agreement with other semicrystalline polymers [13,14,15,16,17,18]. There is a shift of glass transition temperature (T_g_) to a lower value; this is due to the physical aging of polymers, where the crystalline part of polymer increases with the addition of AM and the amorphous part, which is related to a decrease in T_g_ [12]. Pure PLA exhibits an exothermic maximum cold crystallization peak at 131 °C, its cold crystallization enthalpy is around 27.97 J/g, and its present of crystallinity is 30%. For PLA/AM composites, there is a dramatic decrease in the cold crystallization temperature of pure PLA from 131 °C to about 90 °C and within a narrow cold crystallization peak in the presence of 0.5 to 2% of AM. The percent of crystallinity of PLA with the addition of 2 wt% AM is 50%, which suggests that AM is efficient for enhancing the crystallization process of PLA. This result is agreement with the X-ray results. Endothermic melting lines were detected for PLA in the PLA/AM composite. The maximum endothermic melting temperature of PLA decreased with the addition of AM, which is an efficient nucleating agent, whereas the addition of nucleating agents to the polymer can decrease its melting temperature by promoting the formation of smaller crystalline structures. These agents act as sites for nucleation, accelerating the crystallization process. Smaller crystals have a higher surface energy, which leads to a decrease in the overall melting temperature of the material. Double melting lines were estimated for all tested [PLA/AM] composites. A similar behaviour was detected by other authors that investigated PLA composites [10,11,12,13], and this occurrence indicates the melting manner of crystals through various steps of purification. Note that the obtained data for the maximum melting line of the PLA in PLA/AM composites has no relation with structure or composition.

#### 3.8.2. Non-Isothermal Cold Crystallization Kinetics of PLA/AM Composites

**Figure 10:**
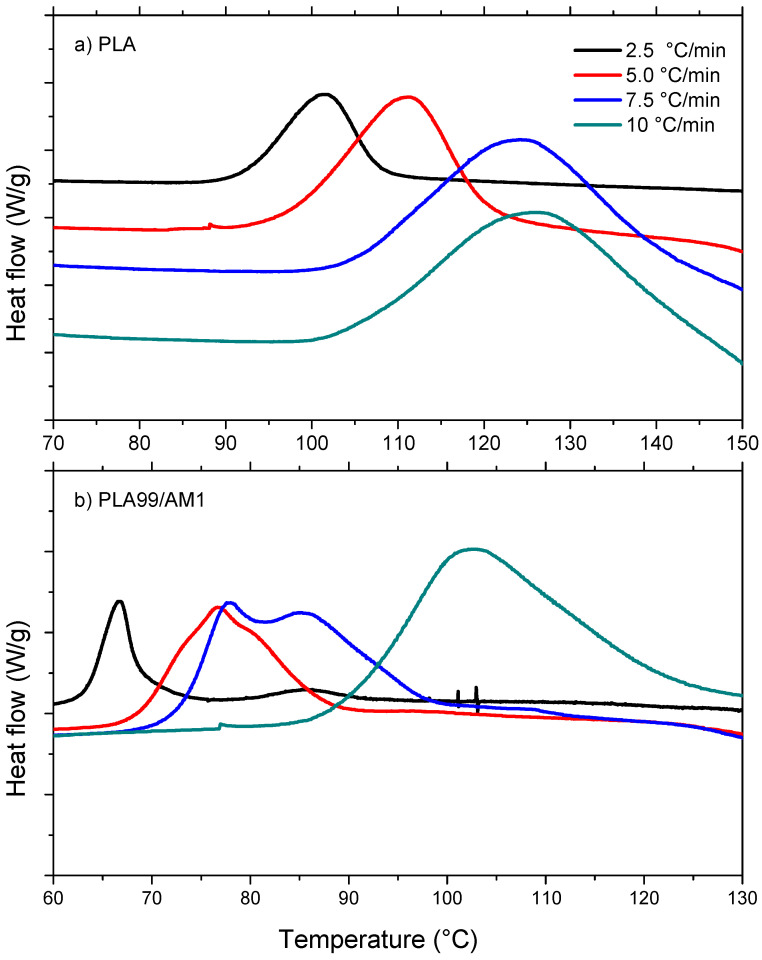
DSC curves for (**a**) PLA and (**b**) PLA99/AM1.

At various heating rates, the cold crystallization temperature (T_cc_) was found to be in the range of 101–127 °C for pure PLA, whereas the PLA99/AM1 composite showed T_cc_ in the range of 67–102 °C under the same heating conditions, as shown in Figure 10 and Table 4. Both PLA and PLA99/AM1 composites exhibited a rise in T_cc_ with a rise in the heating rate. This is because cold crystallization takes more time when heating rates are higher. The values of the maximum crystallization temperatures T_cc_ of PLA99/AM1 were dramatically decreased than those of pure PLA at a given heating rate. This finding suggests that PLA99/AM1 crystallized earlier than PLA alone. The ideal ΔH_CC_ and X_CC_% were obtained when they were increased with the heating rate, and the percentages increased after the addition of 1% AM. Thus, nucleating agents such as (AM) can significantly enhance the crystallization behaviour of polymers, but there is a limit to their effectiveness, beyond which further increases in concentration may yield diminishing returns. Understanding and optimizing the interactions between nucleating agents and polymers is essential for maximizing their benefits and achieving desired crystallization outcomes.

#### 3.8.3. Relative Crystallinity

**Figure 11:**
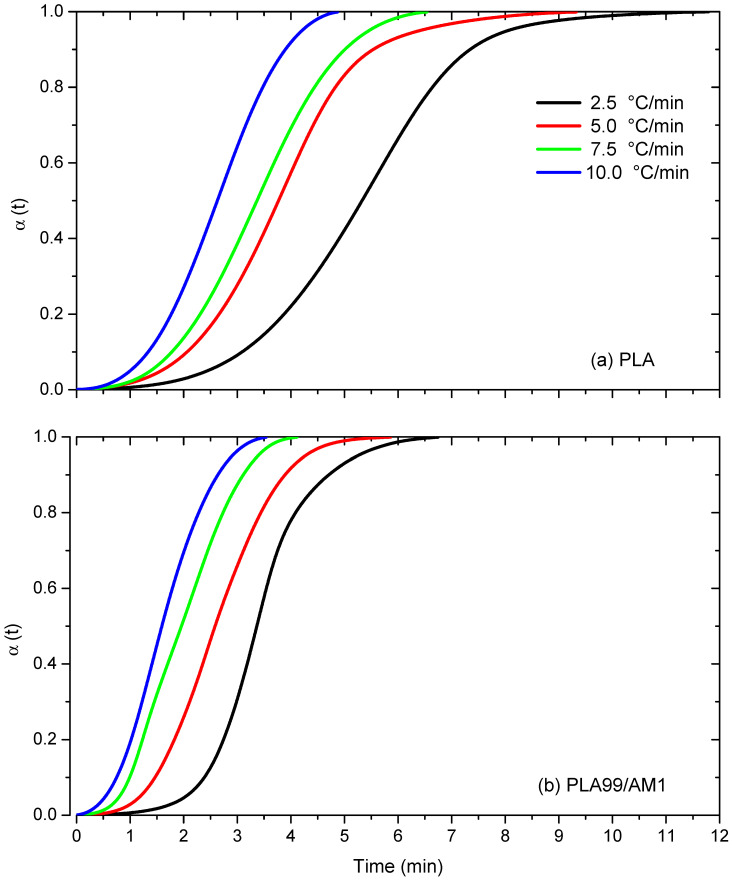
Relative crystallinity curves for (**a**) PLA and (**b**) its composite.

The formula for the proportional level of crystallinity, α(T), in relation to crystallization temperature T is as follows [41]:

(2) $$\alpha (T)=\int_{T_{0}}^{T}(dH_{\mathrm{CC}}/dT)dT/\int_{T_{0}}^{T_{\infty }}(dH_{\mathrm{CC}}/dT)dT$$

where dH_CC_ is the enthalpy of cold crystallization emitted within an infinitesimal temperature range dT, and T_0_ and T_∞_ denote the crystallization onset and end temperatures, respectively. Figure 11 illustrates the relationship between the crystallization temperature T and the relative degree of crystallinity α(T) at various heating rates. The relationship below can be used to convert the data of α(T) into α(t), as follows:

t = (T_0_ − T)/Φ (3)

where T is the temperature at crystallization time t.

The non-isothermal crystallization kinetics of PLA samples were reported using the modified Avrami model [44] in the literature [45,46,47]. According to this model, the crystallization time (t) and the relative degree of crystallinity α(t) are associated as follows:

α (t) = 1 − exp (kt^n^) OR ln[−ln(1 − α(t)] = ln k_t_ − n ln t (4)

According to Jeziorny, the heating rate (Φ) should be utilised to properly rectify the k_t_ values [48], as follows:

(5) $$\ln(k_{c})=\frac{\ln(k_{t})}{\Phi }$$

The reason for this correction is that in non-isothermal crystallization, the temperature changes instantly, so the calculated values of the Avrami exponent (n) and the non-isothermal crystallization rate constant (k_t_) do not represent the same physical parameters as they do in isothermal crystallization [49].

The non-isothermal cold crystallization of PLA and PLA/AM composites can be integrated using Equation (2) to calculate the relative crystallinity (α(t)) of each material as a function of time (t). For PLA and PLA99/AM1 composites, relative crystallinity curves obtained at different heating rates (2.5, 5, 7.5, and 10 °C/min) are displayed as a function of crystallization time in Figure 11. The occurrence of spherulite impinging at a later stage of the cold crystallization process is indicated by the sigmoidal form of the relative crystallinity curves for both PLA and PLA99/AM1 composites. As Figure 11 illustrates, lag effects are minimal for both PLA and PLA99/1AM composites at faster heating rates when compared to slower heating regimes.

##### Half-Time of Crystallization

The time required to complete 50% of the crystallization process is known as the crystallization half-time. Calculating the crystallization half-time requires an understanding of the kinetics of the process. Table 5 displays the PLA t_1/2_ values. It indicates that the t_1/2_ values show a decreasing trend with increased heating rates, indicating a faster crystallization rate for PLA. These results demonstrate how the use of AM has enhanced PLA’s cold crystallization procedure. Furthermore, as shown in Table 5, secondary crystallization is documented for all samples, including PLA and PLA99/1AM composites, which may be inferred from greater t_1/2_ values at lower heating rates.

**Figure 12:**
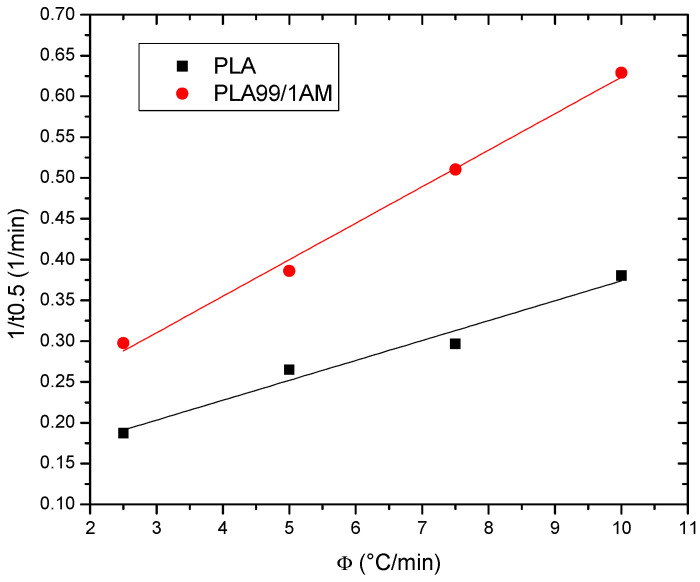
Crystallization rate parameter (CRP) for PLA and its composite.

The crystallization rate parameter (CRP), another metric, was employed to compare the crystallization rates of PLA and its composites. The crystallization rate of polymers is represented by the CRP. The CRP values in the plots of 1/t_1/2_ vs. the heating rate can be calculated from the slopes of the straight lines, with a higher slope resulting from a faster crystallization rate. The associated plots of samples are shown in Figure 12, and Table 5 lists the detailed CRP values.

##### Avrami Model

**Figure 13:**
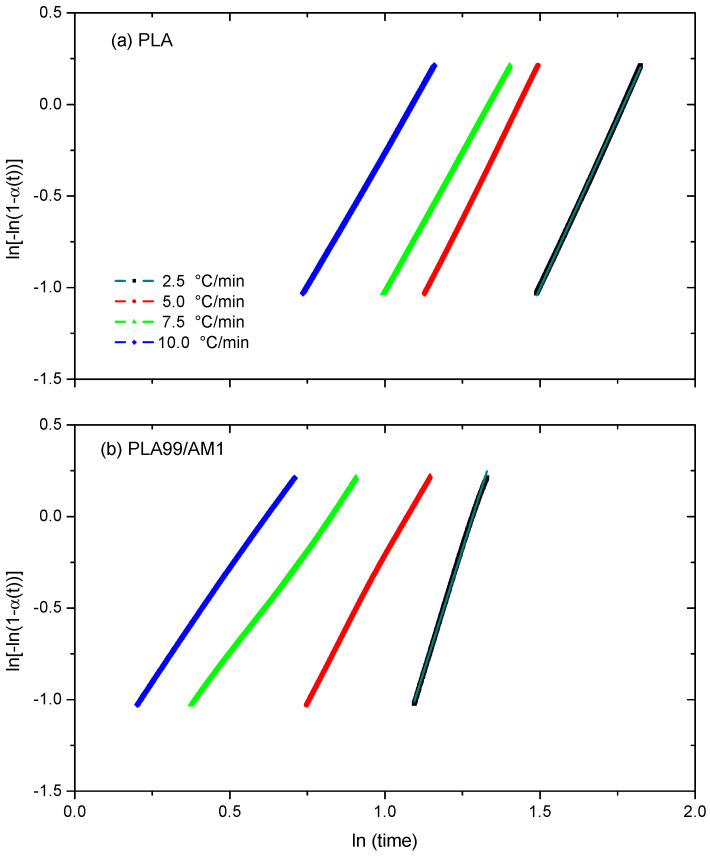
Non-isothermal transformation described by using the Avrami equation as a plot of ln(−ln(1 − α)) against ln(time) for (**a**) PLA and (**b**) its composites.

The simplest Avrami kinetic model is used in this study to establish the non-isothermal cold crystallization kinetic parameters for PLA and PLA99/AM1 composite, which are then stated by Equation (4). In Figure 13, where ln[ln(1(t))] is plotted as a function of ln t, the Avrami plots for PLA and PLA99/AM1 composite formed at various heating rates are displayed. Avrami plots are indicated by the relative crystallinity curves for neat PLA and PLA99/AM1 composites in Figure 13. Figure 13 makes it clear that all of the plots show good linearity to one another. This shows that the theory appears to be helpful for analysing the cold crystallization process of PLA and PLA99/AM1 composite systems. Table 5 lists the Avrami parameters (kc and n) for PLA and PLA99/AM1 composites. The n values for PLA are inferred to be between 2.4 and 3.7, which is consistent with the cited literature [50,51,52,53]. The n values, according to the results, lie between 3.9 and 2.6. This result indicates that the three-dimensional spherulitic development of the pure PLA and its samples is heterogeneous. The same outcomes were also reported by [49,50,51,52]. The results of t0.5 and CRP are corroborated by the values of kc in Table 5.

## 4. Conclusions

In the polylactic acid/Co_0.9_Cu_0.1_Fe_2_O_4_ ceramic magnetic nanocomposites, while nanoparticles (AM) were present, the POM detected a significant number of little PLA spherulites, and these increased because the AM contents increased. X-ray diffraction proved that the nanoparticles did not change the PLA’s crystal structure. From DSC and TGA results, the presence of AM nanoparticles leads to the following trends:

(i) Shift of glass transition temperature (T_g_) to a lower value.
(ii) Shift of crystallization temperature to a lower value.
(iii) Shift of melting temperature to a lower value.
(iv) A rise in the rate of crystallization.
(v) Increase in the percent of crystallinity.
(vi) Decrease in thermal stability of PLA samples.

The Avrami model was used to examine the non-isothermal cold crystallization data of PLA/AM acquired using DSC. For PLA and PLA99/AM1, Avrami’s technique can accurately represent the experimental data. The addition of 1 mass AM% was more effective than the pure PLA, according to the Avrami rate constant and crystallization time measurements. A 3D spheroidal development mechanism is suggested to be present by the variety in modified Avrami defences found in pure PLA and PLA/AM composites. All the obtained results revealed that AM is an effective PLA nucleating agent.

## Figures and Tables

**Table 1:** PLA and its composites.

Sample Name	PLA/Spinel Ferrites Co_0.9_Cu_0.1_Fe_2_O_4_ (AM)
PLA	100:0
PLA99.5/AM0.5	99.5:0.5
PLA99/AM1	99:1
PLA98/AM2	98:2

**Table 2:** Thermal stability parameters of PLA and PLA/AM composites.

Name of PLA Samples	PLA Phase	
	T_onset_	T_end_
**PLA**	334	371
**PLA99.5/AM0.5**	269	342
**PLA99/AM1**	281	356
**PLA98/AM2**	261	335

**Table 3:** Thermal parameters of PLA/AM composites from DSC 2nd heating curves.

Samples	T_g_ (°C)	ΔC_p_ (J/g °C)	T_cc_ (°C)	ΔH_cc_ (J/g °C)	T_m_ (°C)	ΔH_m_ (J/g °C)	X_c_ (%)
PLA	59	0.55	131	27.97	166	33.43	29.88
PLA99.5/AM0.5	46	0.54	96	40.78	147–159	46.01	43.79
PLA99/AM1	39	0.48	90	40.80	139–155	42.38	44.03
PLA98/AM2	55	0.48	108	46.18	158–165	54.75	50.34

**Table 4:** The crystallization transition temperature T_cc_ and the ∆H_cc_ at each heating rate for two samples under study.

Samples	Heating Rate (°C/min)	T_cc_ (°C)	∆H_cc_ (J/g °C)	X_c_ (%)
**PLA**	2.5	33.7	31.55	101
	5	36.4	34.1	111
	7.5	42.3	39.51	125
	10	35.8	33.51	127
**PLA99/AM1**	2.5	28.5	26.41	67
	5	41.1	38.04	77
	7.5	43.7	40.46	78–85
	10	55.6	51.53	102

**Table 5:** The slop (n), rate constant (k), and R-squared for each sample at each heating rate.

Sample	Φ	t_0.5_ Min	CRP (1/°C)	n	k	R^2^
**PLA**	2.5	5.33	0.024	3.71	0.064	**1.00**
	5	3.77		3.42	0.375	**1.00**
	7.5	3.37		3.06	0.581	**1.00**
	10	2.63		2.94	0.726	**1.00**
**PLA99/AM1**	2.5	3.36	0.045	3.37	0.072	**1.00**
	5	2.59		3.12	0.512	**1.00**
	7.5	1.96		2.29	0.777	**1.00**
	10	1.59		2.44	0.86	**1.00**

## Data Availability

The raw data supporting the conclusions of this article will be made available by the authors on request.

## Supplementary Materials

The following supporting information can be downloaded at: https://www.mdpi.com/article/10.3390/polym16091190/s1, Figure S1: X-ray diffraction (XRD) lines of the investigated Cu_0.1_Co_0.9_Fe_2_O_4_ nanoparticles; Figure S2: The infrared transmittance spectra of the investigated Cu_0.1_Co_0.9_Fe_2_O_4_ nanoparticles; Table S1: EDXS analytical study for the compositional elements in the nuclear % (at. %) of [Cu_0.1_Co_0.9_Fe_2_O_4_] mixed nano spinel ferrites.

## References

[1] Mohamed W.S., Abu-Dief A.M. (2020). Impact of rare earth europium (RE-Eu^3+^) ions substitution on microstructural, optical and magnetic properties of CoFe_2−x_Eu_x_O_4_ nanosystems. Ceram. Int..

[2] Marzouk A.A., Abu-Dief A.M., Abdelhamid A.A. (2018). Hydrothermal preparation and characterization of ZnFe_2_O_4_ magnetic nanoparticles as an efficient heterogeneous catalyst for the synthesis of multi-substituted imidazoles and study of their anti-inflammatory activity. Appl. Organomet. Chem..

[3] Balaji A.B., Pakalapati H., Khalid M., Walvekar R., Siddiqui H. (2017). Natural and Synthetic Biocompatible and Biodegradable Polymers.

[4] Zhong Y., Godwin P., Jin Y., Xiao H. (2019). Biodegradable polymers and green-based antimicrobial packaging materials: A mini-review. Adv. Ind. Eng. Polym. Res..

[5] Perera K.Y., Jaiswal A.K., Jaiswal S. (2023). Biopolymer-Based Sustainable Food Packaging Materials: Challenges, Solutions, and Applications. Foods.

[6] Doppalapudi S., Jain A., Khan W., Domb A.J. (2014). Biodegradable polymers—An overview. Polym. Adv. Technol..

[7] Marsilla K.I.K., Verbeek C.J.R. (2017). Crystallization of itaconic anhydride grafted poly(lactic acid) during annealing. J. Appl. Polym. Sci..

[8] Nanthananon P., Seadan M., Pivsa-Art S., Suttiruengwong S. (2015). Enhanced crystallization of poly(lactic acid) through reactive aliphatic bisamide. IOP Conf. Ser. Mater. Sci. Eng..

[9] Saeidlou S., Huneault M.A., Li H., Park C.B. (2012). Poly(lactic acid) crystallization. Prog. Polym. Sci..

[10] Battegazzore D., Bocchini S., Frache A. (2011). Crystallization kinetics of poly(lactic acid)-talc composites. Express Polym. Lett..

[11] Li W., Zhang C., Chi H., Li L., Lan T., Han P., Chen H., Qin Y. (2017). Development of antimicrobial packaging film made from poly(lactic acid) incorporating titanium dioxide and silver nanoparticles. Molecules.

[12] Chu Z., Zhao T., Li L., Fan J., Qin Y. (2017). Characterization of antimicrobial poly(lactic acid)/nano-composite films with silver and zinc oxide nanoparticles. Materials.

[13] Tham W.L., Poh B.T., Mohd Ishak Z.A., Chow W.S. (2014). Thermal behaviors and mechanical properties of halloysite nanotube-reinforced poly(lactic acid) nanocomposites. J. Therm. Anal. Calorim..

[14] Mohammed B.H., Sherwani A.F.H., Faraj R.H., Qadir H.H., Younis K.H. (2021). Mechanical properties and ductility behavior of ultra-high performance fiber reinforced concretes: Effect of low water-to-binder ratios and micro glass fibers. Ain Shams Eng. J..

[15] Wang G., Zhang D., Li B., Wan G., Zhao G., Zhang A. (2019). Strong and thermal-resistance glass fiber-reinforced polylactic acid (PLA) composites enabled by heat treatment. Int. J. Biol. Macromol..

[16] Chen P., Wang W., Wang Y., Yu K., Zhou H., Wang X., Mi J. (2017). Crystallization-induced microcellular foaming of poly(lactic acid) with high volume expansion ratio. Polym. Degrad. Stab..

[17] Wu N., Wang H. (2013). Effect of zinc phenylphosphonate on the crystallization behavior of poly(l-lactide). J. Appl. Polym. Sci..

[18] Harris A.M., Lee E.C. (2008). Improving mechanical performance of injection molded PLA by controlling crystallinity. J. Appl. Polym. Sci..

[19] Xiao H.W., Li P., Ren X., Jiang T., Yeh J. (2010). Isothermal crystallization kinetics and crystal structure of poly(lactic acid): Effect of triphenyl phosphate and talc. J. Appl. Polym. Sci..

[20] Mohamed W.S., Alzaid M., SMAbdelbaky M., Amghouz Z., García-Granda S., MAbu-Dief A. (2019). Impact of Co^2+^ substitution on microstructure and magnetic properties of Co_x_Zn_1−x_Fe_2_O_4_ nanoparticles. Nanomaterials.

[21] Fariñas J.C., Moreno R., Pérez A., García M.A., García-Hernández M., Salvador M.D., Borrell A. (2018). Microwave-assisted solution synthesis, microwave sintering and magnetic properties of cobalt ferrite. J. Eur. Ceram. Soc..

[22] Karbasi M., Maghazeii F., Ghanbari D. (2019). Magnetic investigation of microwave synthesized and thermal stable poly vinyl alcohol-cobalt ferrite nanocomposites. J. Nanostruct..

[23] PAsogekar P.A., Verenkar V. (2019). Structural and magnetic properties of nanosized Co_x_Zn_(1−x)_Fe_2_O_4_ (x = 0.0, 0.5, 1.0) synthesized via autocatalytic thermal decomposition of hydrazinated cobalt zinc ferrous succinate. Ceram. Int..

[24] Sartori K., Choueikani F., Gloter A., Begin-Colin S., Taverna D., Pichon B.P. (2019). Room temperature blocked magnetic nanoparticles based on ferrite promoted by a threestep thermal decomposition process. J. Am. Chem. Soc..

[25] Lavorato G., Alzamora M., Contreras C., Burlandy G., Litterst F.J., Baggio-Saitovitch E. (2019). Internal structure and magnetic properties in cobalt ferrite nanoparticles: Influence of the synthesis method. Part. Part. Syst. Charact..

[26] Sagadevan S., Podder J., Das I., Ebenezar J. (2017). Synthesis and characterization of cobalt ferrite (CoFe_2_O_4_) nanoparticles prepared by hydrothermal method. Recent Trends in Materials Science and Applications.

[27] Gmelin E., Sarge M.S. (1995). Calibration of differential scanning calorimeters. Pure Appl. Chem..

[28] Migliaresi C., Cohn D., De Lollis A., Fambri L. (1991). Dynamic mechanical and calorimetric analysis of compression-molded PLLA of different molecular weights: Effect of thermal treatments. J. Appl. Polym. Sci..

[29] Routray K.L., Saha S., Behera D. (2018). Green synthesis approach for nano sized CoFe_2_O_4_through aloe vera mediated sol-gel auto combustion method for high frequency devices. Mater. Chem. Phys..

[30] Abd El Aleem M., El-Remaily A.A., Abu-Dief A.M. (2015). CuFe_2_O_4_ nanoparticles: An efficient heterogeneous magnetically separable catalyst for synthesis of some novel propynyl-1H-imidazoles derivatives. Tetrahedron.

[31] Abu-Dief A.M., Abdelbaky M.S., Martínez-Blanco D., Amghouz Z., García-Granda S. (2016). Effect of chromium substitution on the structural and magnetic properties of nanocrystalline zinc ferrite. Mater. Chem. Phys..

[32] Abu-Dief A.M., Mohamed W.S. (2017). α-Bi_2_O_3_ nanorods: Synthesis, characterization and UV-photocatalytic activity. Mater. Res. Express.

[33] Alahmadi M., Alsaedi W.H., Mohamed W.S., Hassan H.M., Ezzeldien M., Abu-Dief A.M. (2023). Development of Bi_2_O_3_/MoSe_2_ mixed nanostructures for photocatalytic degradation of methylene blue dye. J. Taibah Univ. Sci..

[34] Tahir M.B., Iqbal T., Hassan A., Ghazal S. (2017). Wet chemical Co-precipitation synthesis of nickel ferrite nanoparticles and their characterization. J. Inorg. Organomet. Polym. Mater..

[35] Mehran E., Shayesteh S.F., Sheykhan M. (2016). Structural and magnetic properties of turmeric functionalized CoFe_2_O_4_ nanocomposite powder. Chin. Phys. B.

[36] Thakur P., Sharma R., Sharma V., Barman P.B., Kumar M., Barman D., Katyal S.C., Sharma P. (2017). Gd doped Mn-Zn soft ferrite nanoparticles: Superparamagnetism and its correlation with other physical properties. J. Magn. Magn. Mater..

[37] Waldron R.D. (1955). Infrared Spectra of Ferrites. Phys. Rev..

[38] Bakhshi H., Shokuhfar A., Afghahi S.S.S. (2015). Structural, magnetic and Raman study of CoFe_2_O_4_@C core–shell nanoparticles. Ceram. Int..

[39] Sharma R., Thakur P., Kumar M., Thakur N., Negi N.S., Sharma P., Sharma V. (2016). Improvement in magnetic behaviour of cobalt doped magnesium zinc nano-ferrites via co-precipitation route. J. Alloys Compd..

[40] Vinothkumar P., Manoharan C., Shanmugapriya B., Bououdina M. (2019). Effect of reaction time on structural, morphological, optical and photocatalytic properties of copper oxide (CuO) nanostructures. J. Mater. Sci. Mater. Electron..

[41] Abu-Dief A.M., Nassar I.F., Elsayed W.H. (2016). Magnetic NiFe_2_O_4_ nanoparticles: Efficient, heterogeneous and reusable catalyst for synthesis of acetylferrocene chalcones and their anti-tumour activity. Appl. Organomet. Chem..

[42] Aleem Ali El-Remaily M.A.E., Abu-Dief A.M., El-Khatib R.M. (2016). A robust synthesis and characterization of superparamagnetic CoFe_2_O_4_ nanoparticles as an efficient and reusable catalyst for green synthesis of some heterocyclic rings. Appl. Organomet. Chem..

[43] Mohamed W.S., Hadia N.M.A., Alzaid M., Abu-Dief A.M. (2022). Impact of Cu^2+^ cations substitution on structural, morphological, optical and magnetic properties of Co_1-x_Cu_x_Fe_2_O_4_ nanoparticles synthesized by a facile hydrothermal approach. Solid State Sci..

[44] Avrami M. (1940). Kinetics of phase change. II Transformation-time relations for random distribution of nuclei. J. Chem. Phys..

[45] Tang L., Qiu Z. (2016). Enhanced nonisothermal and isothermal cold crystallization kinetics of biodegradable poly(l-lactide) by trisilanolisobutyl-polyhedral oligomeric silsesquioxanes in their nanocomposites. J. Appl. Polym. Sci..

[46] Tri P.N., Domenek S., Guinault A., Sollogoub C. (2013). Crystallization behavior of poly(lactide)/poly(β-hydroxybutyrate)/talc composites. J. Appl. Polym. Sci..

[47] Li C., Dou Q. (2014). Non-isothermal crystallization kinetics and spherulitic morphology of nucleated poly(lactic acid): Effect of dilithium hexahydrophthalate as a novel nucleating agent. Thermochim. Acta.

[48] Jeziorny A. (1978). Parameters characterizing kinetics of nonisothermal crystallization of poly(ethylene-terephthalate) determined by DSC. Polymer.

[49] Li M., Hu D., Wang Y., Shen C. (2010). Nonisothermal Crystallization Kinetics of Poly(lactic acid) Formulations Comprising Talc With Poly(ethylene glycol). Polym. Eng. Sci..

[50] Li C., Dou Q., Bai Z., Lu Q. (2015). Non-isothermal crystallization behaviors and spherulitic morphology of poly(lactic acid) nucleated by a novel nucleating agent. J. Therm. Anal. Calorim..

[51] Wu J., Zou X., Jing B., Dai W. (2015). Effect of Sepiolite on the Crystallization Behavior of Biodegradable Poly(lactic acid) as an Efficient Nucleating Agent. Society.

[52] Shi N., Dou Q. (2015). Non-isothermal cold crystallization kinetics of poly(lactic acid)/poly(butylene adipate-co-terephthalate)/treated calcium carbonate composites. J. Therm. Anal. Calorim..

[53] Xiong J., Gong D.-P., Sun Y.-M., Zhao X.-P. (2019). Effect of sulfur phase transition on polypropylene crystallization. Polym. Technol. Mater..

