# Supplementary material for: Effect of Nano Spinel Ferrites Co0.9Cu0.1Fe2O4 on Non-Isothermal Cold Crystallization Behaviours and Kinetics of Its Composites with Polylactic Acid
Source: Polymers (Basel). 2024 Apr 24;16(9):1190. doi: 10.3390/polym16091190 (PMC11085212; doi:10.3390/polym16091190)
Supplement: Supplementary file 1 [file polymers-16-01190-s001.zip › polymers-2949747-supplementary.pdf]

Effect of Nanospinel Ferrites $Co_{0.9}Cu_{0.1}Fe_2O_4$ on Nonisothermal Cold Crystallization Behaviours and kinetics of its Composites with Poly(Lactic Acid).

Wael H Alsaedi [1], Khulood A. Abu Al-Ola [1], Omaima Alhaddad [1], Zyzafon Albelwe [1], Renad Alawaji [1] and Ahmed M. Abu-Dief* [1,2]

[1] Department of Chemistry, College of Science, Taibah University, 30002 Al-Madinah Al-Munawarah, Saudi Arabia.

[2] Department of Chemistry, Faculty of Science, Sohag University, Sohag 82524, Egypt

Corresponding author e-mail: amamohammed@taibahu.edu.sa (Ahmed M. Abu-Dief)

Supporting information

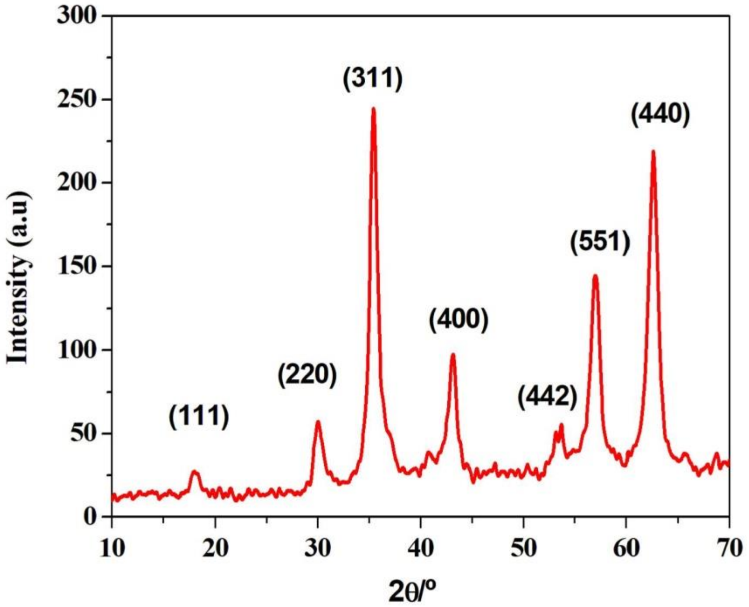

Figure S1. : X-ray diffraction (XRD) lines of the investigated Cu $_{0.1}$ Co $_{0.9}Fe_2O_4$ nanoparticles.

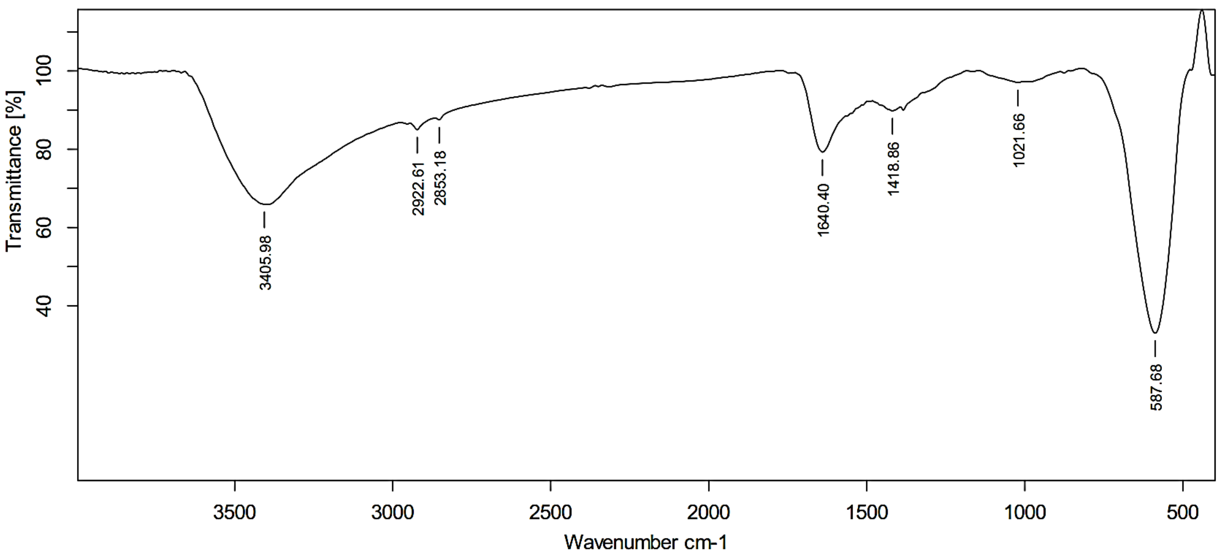

Fig. S2: The infrared transmittance spectra of the investigated $Cu_{0.1}Co_{0.9}Fe_2O_4$ nanoparticles.

Table S1. EDXS analytical study for the compositional elements in the nuclear % (at. %) of [$Cu_{0.1}Co_{0.9}Fe_2O_4$] mixed nano spinel ferrites.

	Elements in atomic percentage (at. %)				Total %
**Element**	Cu	Co	Fe	O	
**Theoretical (Expected)**	1.53	13.56	30.11	54.80	100
**Experimental (Actual)**	1.57	12.67	30.64	55.10	99.98
